# Spermicidal and anti-*Trichomonas vaginalis* activity of Brazilian *Sapindus saponaria*

**DOI:** 10.1186/1472-6882-13-196

**Published:** 2013-07-28

**Authors:** Edilson Damke, Joyce K Tsuzuki, Francieli Chassot, Diógenes AG Cortez, Izabel CP Ferreira, Cristiane SS Mesquita, Vânia RS da-Silva, Terezinha IE Svidzinski, Márcia EL Consolaro

**Affiliations:** 1 Department of Clinical Analysis and Biomedicine, Division of Clinical Cytology, State University of Maringá, Maringá, Paraná 4860, Brazil; 2 Department of Pharmacy, State University of Maringá, Maringá, Paraná, Brazil; 3 Department of Clinical Analysis and Biomedicine, Division of Medical Mycology, State University of Maringá, Maringá, Paraná, Brazil

**Keywords:** *Sapindus saponaria*, Saponins, Spermicidal, Anti-*Trichomonas vaginalis* activity, Contraceptive

## Abstract

**Background:**

*Sapindus saponaria* is used traditionally for curing ulcers, external wounds and inflammations. The spermicidal and anti-*Trichomonas* activity of *S*. *saponaria* and its effect on *Lactobacillus acidophilus* were evaluated.

**Methods:**

Water-ethanol (WE) and butanolic (BE) extracts, as well as a purified sample of saponins (SP) from *S*. *saponaria* were tested for spermicidal and anti-*Trichomonas* activity and for their effect on *L. acidophilus*.

**Results:**

WE, BE and SP immobilized spermatozoa at a minimum effective concentration (MEC) of 2.5 (gram %) for extracts and 1.25 (gram %) for SP. The effective concentrations that caused 50% immobilization of spermatozoa (EC_50_) were 0.5 (gram %) for WE and SP, and 0.1 (gram %) for BE. The compounds were effective against *Trichomonas vaginalis* (Minimum Inhibitory Concentration = 0.156 mg/mL for WE and BE, and 0.078 mg/mL for SP against a clinical strain (CS); and 0.312, 0.156 and 0.078 mg/mL for WE, BE and SP, respectively, against an ATCC strain). In all concentrations tested, the growth of *L. acidophilus* was not reduced.

**Conclusion:**

The *in vitro* study proved the spermicidal and anti-*Trichomonas* activity of *S. saponaria.* Complementary *in vivo* studies should be made for establish the use as a vaginal spermicide, particularly in Brazil and Latin America.

## Background

The sexually transmitted diseases (STDs) are among the most common public-health problems in Brazil and worldwide, and are currently considered the main factor facilitating sexual transmission of HIV/AIDS [1-3]. Most family planning methods, such as oral and injectable hormonal contraceptives, implants, intrauterine devices (IUDs) and sterilization, for example, are effective against unwanted pregnancy, but do not protect against STDs. Safe, effective, acceptable, and self-administered topical preparations with both microbicidal and spermicidal activity are likely to have a major positive impact on reproductive health, especially in areas with a high prevalence of STDs, including HIV infection [4].

To our knowledge, no studies have evaluated the spermicidal activity, against STDs or the effect on the vaginal microbiota for the wingleaf soapberry *Sapindus saponaria*. This is a native plant of Brazil, and belongs to the same family as the Indian soapberry *Sapindus mukorossi*, a native Indian plant for which the saponin fraction isolated from the fruit pericarp has shown very promising spermicidal activity [5,6]. The fruit of *Sapindus saponaria* L. (Sapindaceae), a medium-sized tropical tree, is used by population as soap for washing clothes, and for curing ulcers, external wounds and inflammations [7]. Scientific works has shown antimicrobial activity [8-10], but has been little studied. In a recent study, members of our research group isolated and identified the principal constituents of the n-BuOH saponins, saponins (S1 and S2), and also an acyclic oligoglycoside. The same group also demonstrated excellent inhibitory action *in vitro* and *in vivo* of the water-ethanol (WE) and butanolic (BE) extracts against the yeasts *Candida albicans* and non-*C. albicans* isolated from patients with vulvovaginal candidiasis (VVC). The extracts showed no toxicity to HeLa cervical cells [11,12], signaling the possibility of using this plant as an antifungal agent in this pathology.

A nonionic detergent, nonoxynol-9 (N-9) is widely used as a spermicidal compound. It dissolves the lipid components in the cell membrane of spermatozoa and causes their death or inactivation. N-9 also disrupts the membrane of bacteria, viruses and epithelial cells. Its *in vitro* activity against HIV and other STDs, reported in the past [13], has not been confirmed in more recent clinical trials. These new findings eliminated the possibility of a role for N-9 in HIV prevention. In addition, a number of studies have indicated that N-9 and other nonionic detergents are potent *in vitro* inhibitors of *Lactobacillus* species native to the vagina [14-16]. Consequently, repeated use of N-9 containing spermicides is likely to increase the susceptibility of the vagina to STDs including AIDS. In contrast, some studies indicate that saponins of *Sapindus mukorossi* are far less toxic to *Lactobacillus* species compared to N-9 [17]; show potent microbicidal activity against *Trichomonas vaginalis*[18], *Neisseria gonorrhoeae, Escherichia coli*, and HIV-1; and prevent the transmission of the herpes simplex virus and *Chlamydia trachomatis*[19].

The present study evaluated the spermicidal and anti-*Trichomonas* activity of WE and BE extracts, as well as the purified sample of saponins (SP) of *Sapindus saponaria* and their effects on *Lactobacillus acidophilus*, a common member of the vaginal microbiota.

## Methods

### Plant and extracts/saponins obtainment

Dry pericarps of the fruits of *S. saponaria* were collected on the campus of the State University of Maringá, Paraná, Brazil (UEM). The plant was identified by staff members of the UEM Department of Botany, and an exsiccate was deposited in the Herbarium of this institution (HUM 11710).

To obtain the WE extract, dried pericarps of the fruits (450.0 g) of *S. saponaria* were ground and extracted with EtOH:H_2_O (9:1) at room temperature, by dynamic maceration with constant mechanical stirring. Extraction was carried out in an amber flask, maintained at ambient temperature, for six consecutive days, 6 h per day. The extract was concentrated under low pressure in a rotary evaporator, at a temperature of 40°C. After elimination of the solvent, the extract was frozen in liquid nitrogen and lyophilized in a Martin Christ Alpha 1–2 freeze dryer. The lyophilized extract was stored in a closed amber plastic flask and kept frozen. The WE of the pericarp (50.15 g) was chromatographed in a column (ji = 4.0 cm) of silica gel 60 (Merck, Darmstadt, Germany), and eluted with solvents of increasing polarity including hexane, dichloromethane, ethyl acetate, and methanol (Merck, Darmstadt, Germany). The solvents were evaporated at a temperature of 40°C, frozen in liquid nitrogen, and lyophilized in a Martin Christ Alpha 1–2 freeze dryer. The lyophilized dichloromethane, hexane, ethyl acetate, and methanol fractions were stored in closed containers and kept frozen.

To obtain the BE extract, the methanol fraction was suspended in H_2_O and extracted with n-butanol, which after evaporation gave a solid residue (28.9 g) (BE), which was also lyophilized.

To obtain the SP, two treatments were performed on the column with BE, the first on silica gel in ‘flash’ chromatography and the second in a silica-gel column with increasing polarity solvents, to obtain two sesquiterpene saponins and also one acyclic oligoglycoside. The structures were established by spectroscopic methods (^1^H and ^13^C NMR, HSQC, HMBC, and ESI/MS) and by comparing them with literature data [8,10].

### Spermicidal activity

The spermicidal activity was determinate by the minimum effective concentration (MEC) of each compound that causes total immobilization of spermatozoa, indicating not viability, the effective concentration of the compounds that causes 50% immobilization of spermatozoa (EC_50_), supravital staining and hypo-osmotic swelling test.

#### Chemicals and plant components

WE and BE extracts, and SP obtained from *S. saponaria* were tested. N-9 (Preserv® (2% - 20 mg/g) – Blausiegel, positive control) was used as the spermicidal solution. The lyophilized and frozen extracts of *S. saponaria* were dissolved in sterile distilled water just before the experiments. All other chemicals and biochemicals were purchased from Sigma-Aldrich, USA.

#### Semen samples

Fresh human semen samples collected by masturbation were obtained from healthy male donors above 21 (mean = 35.3 ± 2.1) years old. The samples were allowed to liquefy at 37°C for 30 min. The volume, pH, viscosity and morphology of the semen were determined as per World Health Organization guidelines [20]. Semen samples with a spermatozoa count of >60 million per mL, >65% motility, >60% normal physiology, and a normal pH (7.4–8.0), viscosity and volume were used for tests. The analyses were made by the Carl Zeiss PrimoStar (Gottingen, Germany) optical microscope. This research was approved by the Committee for Ethics in Research Involving Humans at the State University of Maringá, Paraná, Brazil (reports No. 132.777/2012) in compliance with the Helsinki Declaration and each male donors involved had signed the consent form.

#### MEC and EC_50_ determination

The MEC of each compound that causes total immobilization of spermatozoa, indicating not viability, was determined by the Sander-Cramer assay [21]. WE, BE and SP of *S. saponaria* were tested at an initial concentration of 10 mg/ml and diluted in hemolysis tubes at the ratio of 1:2 to 1:32 with sterile saline. Sterile saline solution was used for the negative control. Briefly, 0.1 mL of liquefied semen was added to 0.4 mL of spermicidal solution, N-9 or saline and vortexed for 10 s. A wet mount was immediately prepared on a glass slide and examined under an optic microscope. The weakest dilution that completely immobilized all the spermatozoa in 20 s was recorded as MEC in gram % (w/v). This was confirmed in three individual semen samples and five fields of view.

The EC_50_ was determined in a similar manner using serial dilutions (1:5 ratio) of spermicidal solutions at MEC. The weakest dilution that inhibited spermatozoa motility to ~ 50% of the control (sterile saline only) in gram % (w/v) was recorded as EC_50_. This was confirmed in three individual samples and five fields.

#### Supravital staining and hypo-osmotic swelling test

For supravital staining, 10 μL of MEC and EC_50_ tubes was added to new hemolysis tubes containing eosin – nigrosin (1:2). The samples were mixed and a thin smear was prepared on a glass slide and heat-dried. The dead spermatozoa show positive red staining with eosin and the live spermatozoa not staining, show a white color in contrast with the nigrosin background dye. The numbers of unstained and stained spermatozoa were counted in a total of 200 sperm [20].

For the hypo-osmotic swelling test, 0.1 mL of semen was treated with 0.5 mL of sterile saline (control) or spermicidal solution (at MEC) for 1 min/37°C. After centrifugation at 1000 rpm for 5 min, the sperm pellet was treated with 0.5 mL of hypo-osmotic solution (75 mM fructose, 25 mM sodium citrate) for 30 min/37°C. The number of spermatozoa exhibiting characteristic swelling or tail coiling (live) was counted for a total of 200 spermatozoa under an optical microscope.

### Anti-*Trichomonas* activity

#### Chemicals and trophozoites

The *T. vaginalis* cultures were a clinical strain (CS) and the ATCC strain. WE, BE and SP of *S. saponaria* were tested for anti-*Trichomonas* activity. The lyophilized and frozen extracts and SP were dissolved in sterile distilled water just before the experiments. TYM- Trypticase-Yeast extract-Maltose culture medium, supplemented by fetal calf serum, vitamin mixture, penicillin–streptomycin mixture, JC-1 (1,10,3,30-tetraethyl benzimidazole carbocyanines iodine), CCCP-1 (carbonyl cyanide m-chlorophenylhydrazone), proteinase K and dimethylsulfoxide (DMSO) were purchased from Sigma-Aldrich, USA.

#### *T. vaginalis* culture

The trophozoites were grown for 48 h in standard TYM medium (pH6.8) supplemented with 10% FCS, vitamin mixture and 100 U/mL penicillin/streptomycin mixture at 37°C in 15 mL screw-stoppered glass tubes [18]. Then, a suspension was prepared containing 1.0 × 10^5^ to 5.0 × 10^5^ trophozoites/mL, in sterile distilled water, determined in a Neubauer chamber.

#### Susceptibility assay

Susceptibility of *T. vaginalis* was tested as described [22]. Minimum inhibitory concentration of the extracts or SP at which all cells were found dead was considered as its MIC [18]. To evaluate the anti-*Trichomonas* activity and determine the MIC, the WE, BE and SP of *S. saponaria* were used in an initial concentration of 10 mg/mL and diluted in hemolysis tubes at the rate of 1:2 to 1:32 with sterile saline. Briefly, 1.5 mL of the suspension with trophozoites (CS and ATCC strains) was incubated in the presence of serially diluted WE, BE and SP (1.5 mL) in TYM culture medium (1.5 mL) at 35°C/24 h. A tube without added trophozoites was used as negative control, and a tube without added extracts or SP was used as positive control. Cells were checked for viability under the optical microscope.

### The effect on *Lactobacillus acidophilus*

#### Chemicals and microorganisms

Rogosa SL agar (Sigma-Aldrich, USA) and *L. acidophilus* (kindly supplied by the Oswaldo Cruz Institute Foundation, Rio de Janeiro, Brazil) were used. WE, BE and SP of *S. saponaria* were evaluated for their effect on *L. acidophilus*. The lyophilized and frozen extracts and SP were dissolved in sterile distilled water just before the experiments.

### Lactobacillus *culture*

Rogosa SL agar (7.5%; containing 0.132% acetic acid) plates were prepared with (experimental) or without (negative control) the addition of spermicidal agents. Plates containing 10.0, 5.0, 2.5, 1.25, 0.625, 0.310 or 0.165 mg of WE, BE or SP were inoculated with *L. acidophilus* (1 to 5 × 10^8^ CFU (colony-forming units)/mL) and incubated at 37°C in 5% CO_2_ and 95% air for 72 h. Control plates were inoculated simultaneously and incubated similarly. Number and size of colonies were recorded at the end of the experiment.

### Statistical analysis

All experiments were performed three times. The data are expressed as mean ± SEM and analyzed by one-way analysis of variance. p < 0.05 was considered as the criterion for statistical significance.

## Results

### Plants and components

**Figure 1:**
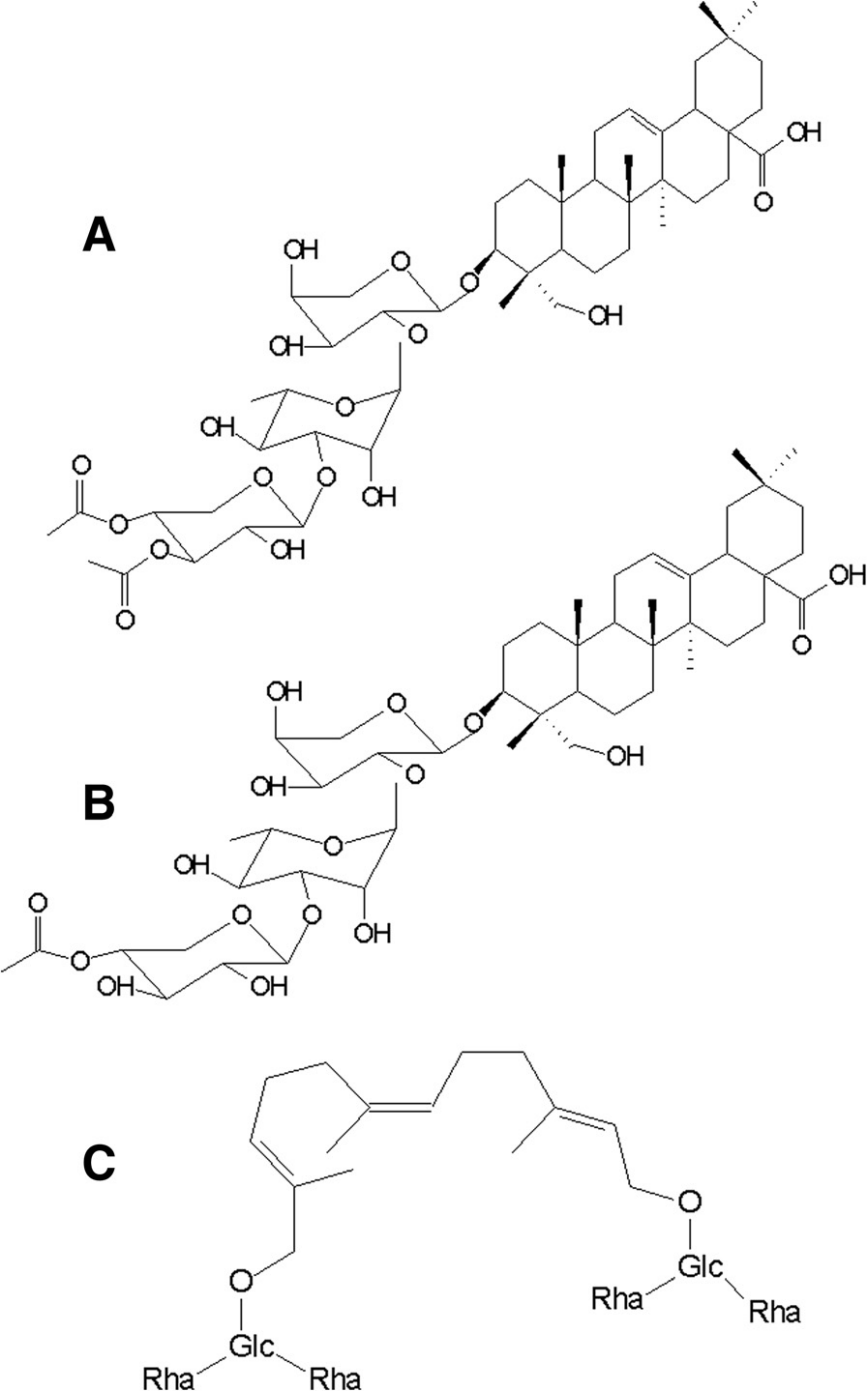
**Chemical components in dry pericarps of the fruits of *S. saponaria.*** Two acetylated triterpene saponins: saponin S1 **(A)**, hederagenin-3-O- (3,4-di-Oacetyl-b-D-xylopyranosyl)-(1®3)-a-L-ramnopyranosyl-(1®2)-a-L-arabinopyranoside; and saponin S2 **(B)**, hederagenin-3-O-(4-O-acetyl-b-D-xylopyranosyl)-(1®3)-a-Lramnopyranosyl-(1®2)-a-L-rabinopyranoside; and also an acyclic oligoglycoside-1 (OGSA-1) **(C)** in water-ethanol (WE) and butanolic (BE) extracts. Saponin S1 **(A)** and saponin S2 **(B)** in saponins (SP) [11].

The presence of two acetylated triterpene saponins was confirmed: saponin S1, hederagenin-3-O- (3,4-di-Oacetyl-b-D-xylopyranosyl)- (1®3)-a-L-ramnopyranosyl- (1®2)-a-L-arabinopyranoside; and saponin S2, hederagenin-3-O-(4-O-acetyl-b-D-xylopyranosyl)-(1®3)-a-Lramnopyranosyl-(1®2)-a-L-rabinopyranoside; and also an acyclic oligoglycoside-1 (OGSA-1) in WE and BE, and S1 and S2; in SP, saponin S1 (A) and saponin S2 (B) as previously described [11] (Figure 1).

### Spermicidal activity

**Figure 2:**
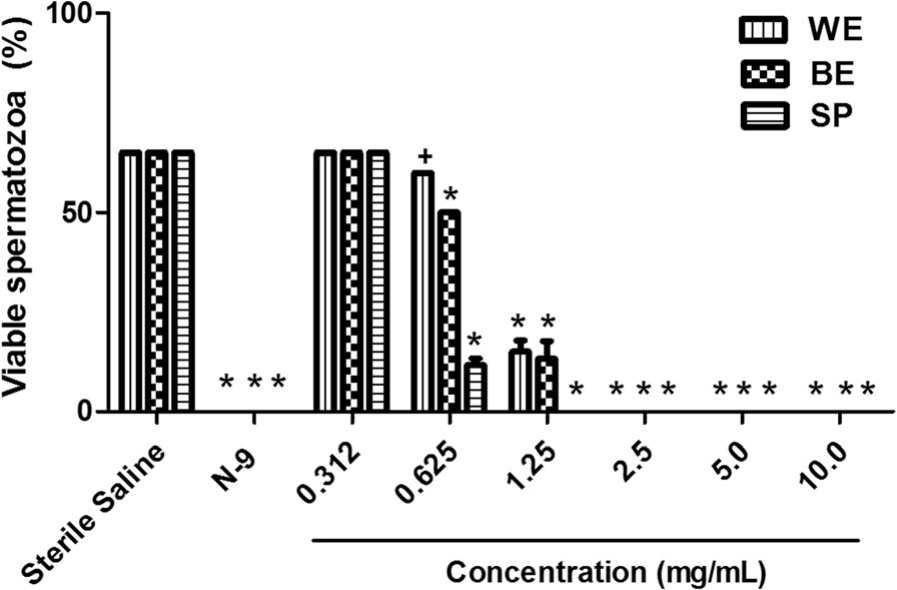
**Effects of water-ethanol (WE) and butanolic (BE) extracts, and individual saponins (SP) of *Sapindus saponaria *on sperm motility in human semen.** Mean ± SEM of percentage of viable spermatozoa (mobile) in different concentrations of WE, BE and SP, in three independent experiments. Nonoxynol-9 (N-9; 2%- 20 mg/g) was used as positive control or spermicidal solution. +p < 0.05, *p < 0.001.

**Figure 3:**
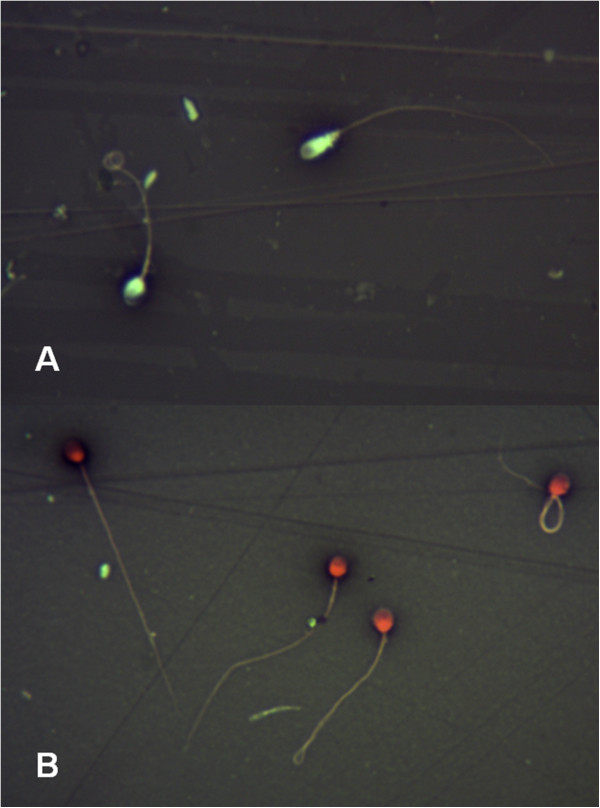
**Sperm imagens of supravital staining containing eosin – nigrosin (1:2).** In the absence of water-ethanol (WE) and butanolic (BE) extracts, and saponins (SP) of *Sapindus saponaria*, the spermatozoa showed negative red staining, indicating live **(A)**. At MEC for WE, BE and SP, 100% of spermatozoa showed positive red staining with eosin, indicating death **(B)**. [magnification, x1000].

The results indicated that the concentrations of 2.5 mg/mL of WE and BE, and 1.25 mg/mL of SP were effective in producing total immobilization, indicating not viability of 100% of spermatozoa (Figure 2). Therefore, the MEC for the extracts was 2.5 (gram %) and for the SP was 1.25 (gram %). There was not viability for N-9, and for the negative control, the viability was 65%. At MEC for WE, BE and SP, 100% of spermatozoa showed positive red staining with eosin, indicating death (Figure 3B), and nearly 100% spermatozoa showed negative hypo-osmotic swelling after treatment with both extracts and SP, indicating complete membrane damage.

**Figure 4:**
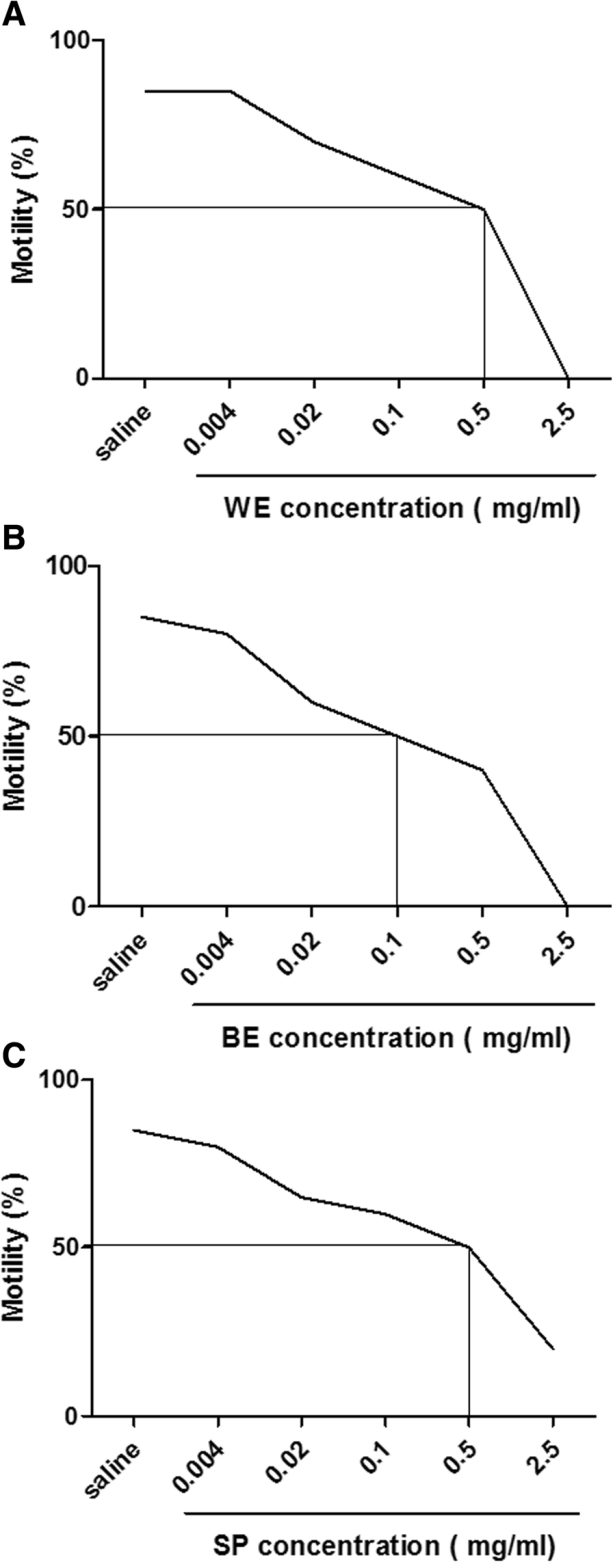
**EC**_**50 **_**determination of the water-ethanol (WE- panel A) and butanolic (BE- panel B) extracts, and saponins (SP- panel C) of *Sapindus saponaria *in human semen.** Each curve represents the concentrations from extracts and SP needed to produce immobility (not viability) of 50% of spermatozoa at MEC, in three independent experiments.

The concentrations needed to produce immobility of 50% of spermatozoa at MEC were 0.5 mg/mL for WE (Figure 4A) and SP (Figure 4C), and 0.1 mg/mL for BE (Figure 4B). Therefore, the EC_50_ was 0.5 (gram %) for WE and SP and 0.1 (gram %) for BE. Supravital staining confirmed that 50% of spermatozoa were dead with EC_50_ values.

### Anti-*Trichomonas* activity

The results showed that both *Trichomonas* strains (CS and ATCC) were inhibited by the WE, BE and SP. The minimum concentrations with no live trophozoites (MIC) for the CS strain were 0.156 mg/mL for WE and BE, and 0.078 mg/mL for SP. The MICs for the ATCC strain were 0.312 mg/mL for WE, 0.156 mg/mL for BE and 0.078 mg/mL for SP (Table 1).

**Table 1:** **Susceptibility of *Trichomonas vaginalis *to water-ethanol (WE) and butanolic (BE) extracts, and saponins (SP) of *Sapindus saponaria***

**Concentration (mg/mL)**	**Viable trophozoites/mL**			**Viable trophozoites/mL**		
	**CS strain**			**ATCC strain**		
	**WE**	**BE**	**SP**	**WE**	**BE**	**SP**
**0.156**	--	--	^--^	7.5 × 10^3^	--	--
**0.078**	2.5 × 10^3^	1.0 × 10^4^	--	1.5 × 10^4^	1.0 × 10^4^	--
**0.039**	2.5 × 10^3^	2.5 × 10^4^	1.5 × 10^4^	1.25 × 10^4^	1.75 × 10^4^	1.25 × 10^4^
**0.019**	2.5 × 10^3^	5.0 × 10^3^	1.0 × 10^4^	6.25 × 10^4^	2.5 × 10^4^	1.0 × 10^4^
**C+**	1.5 × 10^4^	1.25 × 10^4^	1.5 × 10^4^	5.25 × 10^4^	7.5 × 10^3^	5.5 × 10^4^
**C–**	--	--	--	--	--	--

Not found viable trophozoites in concentrations at 0.312 to 10.0 (mg/mL) for extracts or SP.

-- not found viable trophozoites.

C+ positive control - tube without addition of extracts or SP.

C– negative control - tube without addition of trophozoites.

### The effect on *L. acidophilus*

**Figure 5:**
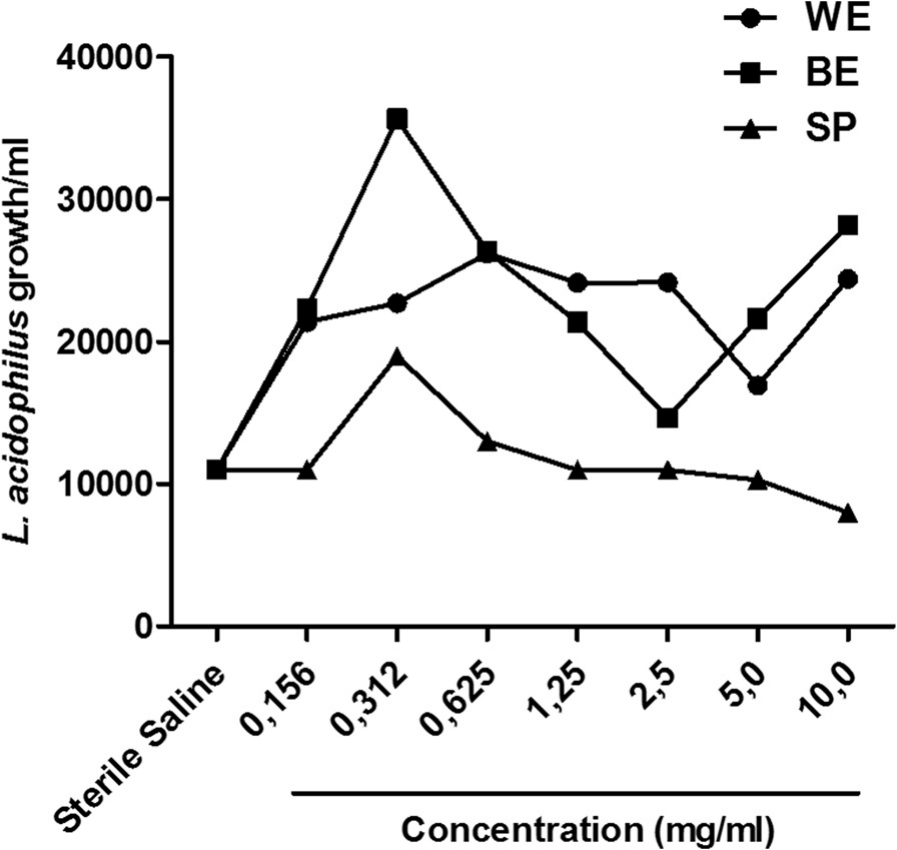
***In vitro *effect the presence of different concentrations of water-ethanol (WE) and butanolic (BE) extracts, and saponins (SP) of *Sapindus saponaria *on *Lactobacillus acidophilus *growth (CFU/mL)*.*** Each curve represents the mean ± SEM of the CFU/mL *in vitro* in three independent experiments.

The growth of *L. acidophilus* was not affected by the two extracts and SP of *S. saponaria* compared with the control (Figure 5).

## Discussion

Considering the need to find spermicidal agents that are more effective in protecting against pregnancy as well as STDs, and are also less toxic, in this study we evaluated the spermicidal and anti-*Trichomonas* activity of WE and BE extracts, as well as the SP of *Sapindus saponaria* and its effect on *Lactobacillus acidophilus*, a common member of the vaginal microbiota.

*S. saponaria* is popularly known as “sabão-de-macaco”, “saboeiro”, “saboneteiro”, “fruta de sabão” and “sabão-de-soldado” [23] and is found in South and Central America, from forests to “cerrado”, a vast tropical savanna ecoregion. In Brazil, it is found from Para State to Rio Grande do Sul State [7,23]. This plant has shown antimicrobial [8,9] and antifungal activities, and no toxicity to HeLa cervical cells [11,12].

In the present experiments, WE, BE and SP of *S. saponaria* were effective for total sperm immobilization (not viability) at MEC of 2.5 (gram %) for extracts and 1.25 (gram %) for SP. At MEC, 100% of spermatozoa showed positive red staining with eosin and negative hypo-osmotic swelling after treatment, indicating complete membrane damage and death. This result is similar to *S. mukorossi*, which is the best-known species of the genus *Sapindus* and is traditionally used in eastern medicine as a spermicide [24]. *S. mukorossi* shows spermicidal activity [6], which is attributed to the presence of saponins [5,25,26]. Similarly, saponins of other plants have been used in contraceptive formulations, either as foaming agents or as spermicidal substances [27,28].

The spermicidal local contraceptives incorporating microbicidal activity can play a significant role in controlling STDs [6]. *T. vaginalis* causes trichomoniasis, which is the most prevalent non-viral human urogenital pathogen [29]. In this study, the compounds inhibited two different strains of *Trichomonas vaginalis.* They were effective against the CS strain (MIC = 0.156 mg/mL for WE and BE, and 0.078 mg/mL for SP) and against the ATCC strain (MIC = 0.312, 0.156 and 0.078 mg/mL for WE, BE and SP, respectively), and the active anti-*Trichomonas* concentrations were lower than its effective spermicidal concentration. This accord with observations on saponins from *S. mukorossi*, which exhibit anti-*Trichomonas* activity at a 10-fold lower concentration than the effective spermicide against human spermatozoa [18]. *T. vaginalis* is inhibited by saponins obtained from other plants [30,31], showing that the anti-*Trichomonas* effect could also be related to the presence of saponins.

*L. acidophilus* is a important organism in the vaginal microbiota, and is responsible for maintaining acidic pH, preventing the growth of potential pathogens [32,33]. The ideal spermicidal agent should preserve the healthier vaginal microflora through retention of *Lactobacillus*. *In vitro* studies have shown that N-9 is detrimental to *Lactobacillus* species [14-16], showing that its use could contribute for increase the incidence of STDs [34-36]. The present study showed that the WE, BE or SP of *S. saponaria* did not alter the growth of *L. acidophilus* colonies, showing that is not toxic to the common vaginal microbiota. This accord with the observations of Ojha et al. [17] who concluded that the saponins are far less toxic compared to N-9.

We acknowledge that *in vivo* studies are needed to completely confirm our results. One of the great challenges of research on the physiopathogenesis of diseases is to match the experimental conditions *in vitro* as much as possible to those *in vivo*, which are often much more complex [37]. However, researchers recognize that these experiments do provide an approximation to *in vivo* conditions [37-39].

## Conclusion

In conclusion, the present study demonstrated that *S. saponaria* or its saponins could be an alternative vaginal spermicide for use in Brazil or Latin America as a whole, either alone or incorporated in condoms or spermicidal creams. *In vivo* studies must be carried out to evaluate its effects and toxicity, and any antimicrobial activity against other microorganisms.

## Abbreviations

WE: Water-ethanol extract of *Sapindus saponaria*; BE: Butanolic extract of *Sapindus saponaria*; SP: Purified sample of saponins from *Sapindus saponaria*; MEC: Minimum effective concentration; EC50: Effective concentration that causes 50% immobilization of spermatozoa; MIC: Minimal inhibitory concentration; CS: Clinical strain; STDs: Sexually transmitted diseases; IUDs: Intrauterine devices; VVC: Vulvovaginal candidiasis; N-9: Nonoxynol-9; UEM: State University of Maringá, Paraná, Brazil; CFU/mL: Colony-forming units per mL.

## Competing interests

The authors declare that they have no competing interests.

## Authors’ contributions

ED carried out the in vitro susceptibility tests and helped to draft the manuscript. JKT prepared the extracts. FC carried out the in vitro susceptibility tests .DAGC and ICPF analyzed the plant components and helped to draft the manuscript. CSSM carried out the in vitro susceptibility tests. TIES helped to conceive the study, participated in its design and coordination, and helped to draft the manuscript. VRSS carried out the in vitro susceptibility tests and helped to draft the manuscript. MELC carried out the cell toxicity analyses, participated in the study coordination, and helped to draft the manuscript. All authors read and approved the final manuscript.

## Pre-publication history

The pre-publication history for this paper can be accessed here:

http://www.biomedcentral.com/1472-6882/13/196/prepub
